# Insulin Resistance in Children

**DOI:** 10.3389/fendo.2019.00342

**Published:** 2019-06-04

**Authors:** Veronica Maria Tagi, Cosimo Giannini, Francesco Chiarelli

**Affiliations:** Department of Pediatrics, University of Chieti, Chieti, Italy

**Keywords:** insulin resistance, children, adolescents, metabolic syndrome, euglycemic clamp, HOMA-IR, QUICKI, WBISI

## Abstract

Insulin resistance (IR) is a pathological condition strongly associated with obesity. However, corticosteroids or growth hormone therapy and genetic diseases may affect insulin sensitivity lifelong. In obese children and adolescents of any age there is an evident association between IR and an increased prevalence of type 2 diabetes (T2D) and other elements contributing to the metabolic syndrome, leading to a higher cardiovascular risk. Therefore, early diagnosis and interventions in the attempt to prevent T2D when glycemia values are still normal is fundamental. The gold standard technique used to evaluate IR is the hyperinsulinemic euglycemic clamp, however it is costly and difficult to perform in clinical and research sets. Therefore, several surrogate markers have been proposed. Although the treatment of insulin resistance in children is firstly targeted to lifestyle interventions, in selected cases the integration of a pharmacological intervention might be taken into consideration. The aim of this review is to present the current knowledge on IR in children, starting with an outline of the recent evidences about the congenital forms of deficiency in insulin functioning and therefore focusing on the physiopathology of IR, its appropriate measurement, consequences, treatment options and prevention strategies.

## Introduction

Insulin resistance (IR) is a shared pathological condition supporting several dysmetabolic status including obesity and type 2 diabetes (T2D), dyslipidemia, atherosclerosis, polycystic ovarian syndrome (PCOS), and non-alcoholic fatty liver disease (NAFLD) (1, 2). An increased degree of IR is common in children and adolescents and is strongly associated with obesity (3). In obese children and adolescents of any age, a strong association between IR and a higher prevalence of the components of the metabolic syndrome (MS) has been observed, therefore a higher cardiovascular risk is predicted in these subjects (4) ^1^,^2^. The pathogenesis of this correlation has been found in the β-cell dysfunction which occurs in obese children and adolescents, due to the increased ectopic fat deposition. Unfortunately, appreciable β-cell destruction may occur before glucose tolerance or fasting glucose levels become impaired (5). Therefore, the advantage to prevent T2D in conditions of euglycemia appears evident. Thus, early recognition of insulin-resistant youths favors both population-based research and clinical practice (6).

The aim of this review is to present the current knowledge on IR in children, starting with an outline of the recent evidences about the congenital forms of deficiency in insulin functioning and therefore focusing on the physiopathology of IR, its appropriate measurement, consequences, treatment options and prevention strategies.

## Definition and Background

IR is a decreased tissue response to insulin-mediated cellular actions (3). It may be due to several causes, including the excess of adipose tissue, which is physiologically insulin resistant.

Although IR is more often related to obesity, also normal-weight children may be affected, suggesting that an increased adiposity is not its unique determinant (7). On the other hand, it has been demonstrated that obesity does not always lead to this pathological condition (8–10).

Other factors responsible for IR are the prolonged use of corticosteroids or growth hormone therapy and some uncommon genetic diseases, due to mutations of the insulin receptor or proteins involved in the transduction of the insulin signal (11). Moreover, puberty is a physiological condition which may be responsible for IR itself (12).

According to euglycemic hyperinsulinemic clamp studies in adult populations, IR is primarily related to the response of skeletal muscle. In fact, it is well-known that the uptake of infused glucose in the muscle is roughly of 75 vs. 2–3% taken by the adipose tissue (9).

The prevalence of obesity is increasing worldwide in children of all ages. In developing countries the prevalence of overweight and obese children aged <5 years previously reported to be 6.1 and 11.7%, was, respectively, drastically increased at an annual rate of 0.5 and 1.1% until 2013 (13). Consequently, a contemporary increasing of the incidence of IR and T2D occurs.

A recent study conducted by Arslanian et al. comparing obese adolescents and adults with impaired glucose tolerance (IGT), showed a greater insulin resistance in adolescents than adults, despite similar degrees of adiposity and glycemic status (14). This finding could give an explanation to the less improvement in insulin sensitivity in response to metformin and faster decline of β-cell function registered in youth than in adults with T2DM.

Different hypotheses have been proposed to understand the physiological mechanisms involved in this substantial different: a stronger impact of obesity on insulin sensitivity in youth, although it has been reported that insulin sensitivity is worsen by aging, a major visceral abdominal fat distribution although total body fat is similar, a superior glucotoxic or inflammatory effect induced by IGT in youth in comparison with adults, or the lower level of HDL in adolescents, considering the theory according to which HDL plays a role in inducing glucose disposal through skeletal muscle.

The clinical presentation of IR is variable and depends on its etiology and severity. The mechanisms responsible for its different signs and symptoms are still unknown. It has been hypothesized that high insulin blood levels, due to the high concentration of glucose, may excessively stimulate specific insulin-dependent pathways, resulting in acanthosis nigricans, ovarian hyperandrogenism (PCOS), lipodystrophy, accelerated or impaired linear growth, autoimmunity, and muscle cramps^3^. The distribution of adipose tissue depends on the type of impaired insulin sensitivity. In fact, central and abdominal obesity, generally associated with the ectopic deposition of fat (e.g., mainly into the muscle and liver) are common, while in most genetic syndromes an ectopic deposition in muscle and liver is associated with a reduced content of fat in the usual fat depot sites.

## Congenital Defects of Insulin Function

In presence of children with severe phenotypes, an inherited alteration of the action of insulin should be suspected and fasting serum insulin should be measured. If insulin is elevated with normal or high blood glucose, additional studies should be directed to look for insulin receptor mutations, circulating anti-insulin receptor antibodies, or other disorders.

Inherited lipodystrophies are rare disorders in which selective loss of adipose tissue and a predisposition to insulin resistance are typical (15). Among them, some manifest at birth, such as congenital generalized lipodystrophy (CGL) and neonatal progeroid syndrome, while others have a later onset, such as familial partial lipodystrophies (FPLD) and mandibulosacral dysplasia manifest later in life. The higher is the extent of body fat loss in these patients, the more severe is the metabolic syndrome by which they are affected. Table 1 lists all subtypes of genetic lipodystrophies classifying them according to their possible inheritance patterns or their possibility to be caused by a de novo heterozygous mutation. The two most prevalent variations are CGL and FPLD. For its complex clinical presentation and severe metabolic complications we will focus on CGL, which has an estimated prevalence of 1 in 10 million.

**Table 1:** Genetic lipodystrophies listed according to their possible inheritance patterns.

**Inherited mutations**		**De novo mutations**
**Autosomal recessive**	**Autosomal dominant**	
Congenital generalized lipodystrophy (Berardinelli–Seip syndrome; AGPAT2, BSCL2, CAV1, PTRF) Mandibuloacral dysplasia (LMNA, ZMPSTE24) Familial partial lipodystrophy (CIDEC, LIPE, WRN, PCYT1A) Autoinflammatory lipodystrophy (JMP/CANDLE syndrome; PSMB8) CGL-like phenotypes (PPARG, FOS)	Familial partial lipodystrophy (LMNA, PPARG, AKT2, PLIN1) Hutchinson–Gilford progeria syndrome (LMNA) Atypical progeroid syndrome (LMNA) Neonatal progeroid syndrome (FBN1, CAV1, and others) Mandibular hypoplasia, deafness, progeroid features (MDP) syndrome (POLD1) SHORT syndrome associated with lipodystrophy (PIK3R1) Keppen–Lubinsky syndrome associated with lipodystrophy (KCNJ6)	Hutchinson–Gilford progeria syndrome; Atypical progeroid syndrome; Mandibular hypoplasia, deafness and progeroid features syndrome.

*CANDLE, chronic atypical neutrophilic dermatosis with lipodystrophy and elevated temperature; CGL, congenital generalized lipodystrophy; JMP, joint contractures, muscle atrophy, microcytic anemia, and panniculitis-induced lipodystrophy; SHORT, short stature, hyperextensibility, hernia, ocular depression, Rieger anomaly, and teething delay*.

Essential diagnostic criteria for CGL are lack of fat tissue involving the whole body and excessive muscular tissue representation at birth or soon thereafter. RMI should be performed to confirm the typical body fat distribution characterizing this syndrome. Molecular diagnosis is the last step which provides certain diagnosis (15). Other clinical features that may be observed are prominent veins, secondary to body fat lack, accelerated growth, due to increased appetite, prominence of umbilicus or umbilical hernias, probably caused by hepatomegaly and/or splenomegaly, fatty liver, which can evolve in fibrosis and cirrhosis requiring liver transplantation in severe cases, acanthosis nigricans, cardio myopathy, focal segmental glomerulosclerosis, mild hirsutism, and clitoromegaly in females, irregular menstrual periods with polycystic ovaries, focal lytic bone lesions, due to the lack of normal bone marrow fat, and advanced bone age. Subjects affected by CGL develop insulin resistance in early childhood and almost half of them manifest diabetes mellitus at puberty. In these patients diabetes mellitus is resistant to ketosis, maybe because of their endogenous hyperinsulinaemia and some of them need very high doses of insulin to control glycaemia, up to 3,000 units per day. Most CGL patients present high triglycerides blood levels from late childhood or adolescence and when it is severe xanthomas and frequent pancreatitis occur, in particular when DM is not well-controlled. Low HDL cholesterol is another frequent finding.

The lack of fat tissue also induces hypoadiponectinemia and hypoleptinaemia, leading to increased appetite and therefore to worsen metabolic complications.

Four types of CGL are known. Type 1 is due to mutations in AGPAT2, encoding lysophosphatidic acid acyltransferase-β, which is involved in triglyceride biosynthesis. Subjects with this subtype of CGL can present typical acromegalic features. Type 2 CGL is secondary to mutations in BSCL2, encoding seipin, a transmembrane protein which seems to play a role in lipid droplet assembly, adipocyte differentiation and fusion of lipid droplets. In subjects with this subtype a higher prevalence of cardiomyopathy and mild mental retardation has been observed. Type 3 CGL is caused by mutations in CAV1, encoding caveolin 1, the main component of caveolae, microdomains of the plasma membrane responsible for promoting the stability and function of lipid droplets, transporting and storing fatty acids and cholesterol and increasing insulin signaling. In this case mechanical adipose tissue and fat in the bone marrow seem preserved (15). In type 4 mutations occur in PTRF, encoding cavin-1, an e substance which is essential for the synthesis of caveolae. In this case lipodystrophy is not severe at birth but might be progressive in childhood. In addition to the classical features, children present congenital myopathy with high blood creatine kinase levels, distal metaphyseal deformation with joint stiffness, pyloric stenosis, atlanto-axial instability, percussion induced, local protracted muscle contractions, and a predisposition to serious arrhythmias which can cause sudden death.

The Seip-Berardinelli syndrome is a rare congenital disorder included in the generalized lipodystrophies, whose estimated prevalence is lower than one case per one million people.

Congenital generalized lipodystrophies are inherited as an autosomal recessive trait and in 95% of reported cases are due to the mutation of *AGPAT2* and *BSCL2* (16). A relevant subcutaneous fat lack is the main sign in early life. Adipose tissue appears very poor in the subcutaneous areas of the abdomen and thorax and in the bone marrow, whereas it is normally represented in the orbits, mouth and tongue, palms and soles, scalp, perineum, and periarticular regions (17). Children affected by Seip-Berardinelli syndrome present with voracious appetite, accelerated growth, increased metabolic rate, and advanced bone age, with a usually normal final height. In addition, these patients may have acanthosis nigricans and hepatic steatosis, which increases the risk to develop cirrhosis, prominent musculature, precocious secondary sexual development, and, in some cases, intellectual impairment (16, 18). IR has an early onset in these patients (19). Studies conducted on the correlation between IR and lipoatrophy do not have an univocal answer concerning an hypothetical altered insulin receptor expression, function and signaling involved in its pathogenesis (20).

Experimental researches (21, 22) suggest that leptin has an important role of leptin and adiponectin in the pathogenesis of IR in Seip-Berardinelli Syndrome, but their mechanism of action in humans is still unknown.

Donohue syndrome, or leprechaunism, is a rare autosomal recessive disorder with a reported incidence of 1 in 4 million live births. It is caused by the mutation of a gene located on chromosome 19p13, altering the correct binding of the insulin receptor to insulin (23). Two milder forms of insulin resistance in which the same mechanism is impaired are Rabson Mendenhall Syndrome and Type A Insulin Resistance syndrome (23).

Donohue syndrome is characterized by severe intrauterine growth retardation and severe failure to thrive, marasmus and malnutrition despite an adequate alimentation in childhood (23). The triad including severe hyperinsulinism, fasting hypoglycaemia, and postprandial hyperglycemia in association with the presence of clinical features involving face (Elfin like pointed chin Microcephaly Low set prominent ears Orbital hypertelorism Broad nose Thick lips Facial hair), skin (Hypertrichosis Acanthosis nigricans Excessive thick, hyperelastic skin Decreased subcutaneous fat Muscle wasting), and other body districts (Large hands and feet (relative to body) Low body weight for age Hypotonia Abdominal distension Reduced lateral thoracic dimensions Hyperplasia of nipples, genitals, other organs) are sufficient for the diagnosis (23).

Low plasma glucose, secondary to the acceleration of fasting metabolism, and reduce response to exogenous insulin in these patients are associated with hypertrophy of the pancreatic islets of Langerhans. Other findings are splenomegaly and hepatomegaly (23), with glycogen and iron accumulation and bile duct cholestasis, enlarged kidneys, nephrocalcinosis, adrenal glands atrophy, delayed bone age, with deformation of metaphysics and epiphysis, and hypertrophic cardiomyopathy, which generally manifests at 1–2 months of age and seems to be caused by supraphysiological hyperinsulinism.

## Physiopathology of Insulin Resistance

Insulin regulates glucose homeostasis acting mostly on hepatic, muscular and fatty tissues. In the hepatic tissue, insulin inhibits gluconeogenesis and glycogenolysis, therefore reducing the production of glucose and induces glycogen storage. In muscular and fatty tissues, it favors the uptake, storage, and use of glucose. Moreover, insulin is responsible for the induction of potassium transport in muscle, of the differentiation of cells into adipocytes, and of the production of androgens by ovaries and retention of sodium by the kidney. Insulin performs all these functions binding with a specific transmembrane protein receptor, which is encoded by a single gene localized on chromosome 19. This interaction induces the processing of a precursor of 1,382 with the final realization of a mature receptor, consisting of two α and two β subunits. The extracellular α subunit is essential for a high-affinity binding of insulin, while the transmembrane part of the β subunit in involved in the transduction of the signal into the cell. Therefore, tyrosine residues of the β subunit located intracellularly undergo phosphorylation, leading to the activation of the intracellular tyrosine kinase (24). Once tyrosine kinase is activated, it phosphorylates tyrosine residues outside the kinase domain of the receptor creating binding sites for signaling proteins with src-homology 2 (SH2) domains, or phosphotyrosine- binding (PTB) domains. The signaling network through which insulin exerts its effects is made of so-called “critical nodes.” Among them, the three best known are the phosphorylation of insulin receptor substrate (IRS) proteins by the activated insulin receptor, the recruitment of class 1A PI3K to the phosphorylated IRS proteins, and therefore the activation of the AKT serine threonine kinases by 2 phosphoinositide-dependent kinases (PDK1 and−2) (25).

In order to understand the pathogenesis of insulin resistance it should considered whether, in addition to the impairment of insulin's glucose-lowering action, its other functions are compromised too. In fact, for a well-known negative feedback loop, the blood glucose concentration regulates insulinemia. For this reason, pathologies involving the only glucose-lowering action determine compensatory increase of blood insulin concentration, determining the exposition of any other less insulin-resistant pathway or tissue to higher insulin action (25). Recent studies on humans have emphasized this concept showing that even severe genetic mutations involving the insulin receptor resulted in hyperglycemia, hyperinsulinemia, ovulatory dysfunction, hyperandrogenism, acanthosis nigricans, and soft tissue overgrowth, but did not show manifestations of impairment of other insulin functions, presenting normal triglycerides, HDL- cholesterol, absence of liver steatosis, and adiponectin within the physiological limits. In a family affected by a defect in the AKT serine threonine kinases, which is more distal in insulin signaling, subjects presented severe liver steatosis, altered lipid profile and low adiponectin concentrations.

Individuals with genetic mutations in C-terminal SH2 domain of class 1A PI3K, located between the insulin receptor and AKT2 in the insulin signaling pathway, have been shown preserved liver fat, lipid profile and plasma adiponectin, although sever insulin resistance is present. Moreover, SHORT syndrome, including short stature, joint hyperextensibility, ocular depression, altered development of the iris (Rieger anomaly), and teething delay (25–27), as well as lipodystrophy, have been found in these patients.

## Methods of Measurement and Diagnosis

The gold standard for the assessment of IR is the hyperinsulinemic euglycemic clamp; however the intravenous glucose tolerance test (IVGTT) and/or the insulin tolerance test (ITT)/insulin suppression test are more frequently used because they are easier to perform (26, 27).

During the hyperinsulinemic-euglycemic clamp insulin is administered intravenously at a constant rate which increases and maintains systemic insulinemia, while a glucose intravenous infusion at variable rates occurs, in order to maintain glucose levels within the normal range.

The glucose infusion rate during the steady state is directly related to insulin sensitivity; in fact, in case of insulin sensitivity, glucose is rapidly consumed by tissues in a condition of hyperinsulinism, therefore high doses of glucose must be infused in order to maintain euglycemia. In contrast, insulin resistant subjects have a low need of glucose infusion to maintain euglycemia since they are characterized by impaired glucose uptake and consumption (28).

The glucose tolerance test (GTT) analyses the effects of exogenous glucose, administered orally, intraperitoneally, or intravenously, on the systemic clearance of glucose. This method is not applicable to individuals with altered pancreatic functioning (28).

Intravenous GTT (IVGTT) consists of measurement of basal insulinemia and afterwards injection of glucose into vein for 3 min, followed by the measurement of blood insulin levels at 1 and 3 min after the injection. IVGTT gives more reliable results, since it avoids variations due to gastro-intestinal factors which occur in case of oral glucose administration^4^.

The insulin tolerance test (ITT) evaluates the systemic glucose clearance in response to intraperitoneal administration of insulin. Severe hypoglycaemia is a frequent adverse effect of ITT and its results may be not reliable in case of systemic counter regulatory responses (28).

The previously mentioned tests are invasive, expensive and complex to use in the daily clinical practice, as well as difficult to perform in population based research studies (29). Therefore, several surrogate tools of measurement have been proposed. In Table 2 the most used methods to evaluate IR in both clinical and research settings are synthesized. The most common surrogate markers are the fasting plasma insulin (FPI), the homeostasis model assessment insulin resistance (HOMA-IR) and the quantitative insulin sensitivity check index (QUICKI), which have been demonstrated to correlate favorably and have been validated in childhood with the hyperinsulinemic-euglycemic clamp (30, 31).

**Table 2:** Main surrogate indexes of Insulin Resistance in children and adolescents.

**Method**	**Parameters**	**Formula**
HOMA1-IR	FPG, FPI	FPG (nmol/l) × FPI (microU/l)/22.5
FPI	FPI	FPI
QUICKI	FPG, FPI	1/logFPI (microU/ml)–logFPG (mg/dl)
FGIR	FPG, FPI	FPG/FPI
HOMA2-IR	FPG, FPI	Computer model
McAuley Index	FPI, triglycerides	2.63–0.28 ln(FPI in mU/l)−0.31 ln(triglycerides in mmol/l)
ISI	BW, FPG, AUCFG, AUCFI, UG	[1.9/6 × BW (kg) × FPG (mmol/l) + 520−1.9/18 × BW × AUCFG (mmol/h·l)—UG (mmol/1.8)]/[AUCFI (pmol/h·l) × BW]
WBISI	FPG, FPI, MG, MI	$\begin{array}{l} \frac{10,000}{\sqrt{\text{FPG in mg}/\text{dl }\times \text{ FPI in microU}/\text{ml})}}\\ \left(\text{MG}\times \text{MI}\right) \end{array}$

*HOMA1-IR, homeostatic model assessment insulin resistance 1; FPI, fasting plasma insulin; QUICKI, quantitative insulin-sensitivity check index; FGIR, fasting glucose/insulin ratio; HOMA2-IR, homeostatic model assessment insulin resistance 2; FPG, fasting plasma glucose; BW, body weight; AUCFG, area under curve for glucose; UG, urinary glucose; AUCFI, area under curve for insulin; MG, mean glucose; MI, mean insulin*.

The FPI is commonly used as surrogate marker of insulin sensitivity by characterizing insulin levels during a fasting state. Although fasting insulin is not considered an adequate method for the evaluation of insulin sensitivity, it may represent a valid index of compensatory hyperinsulinemia and liver insulin metabolism. Moreover, FPI does not always correlate well with IR in pediatric patients (32, 33). In addition, in contrast to other markers such as HOMA-IR, this index does not evaluate insulin concentration in regards to fasting glucose values. HOMA-IR is a paradigm model which allows to determine the IR rate using only the fasting glucose and insulin values. A higher value of HOMA-IR corresponds to a more severe IR (34). Therefore, its great advantage is that it requires a single blood sample. In contrast, obtained data need to be interpreted carefully, especially in subjects with relevant alteration of glucose metabolism (35). It is used to calculate the correlation between steady-state insulinemia and glycemia in order to estimate β-cell function and IR (36). The interaction between fasting glucose and insulin represents an index of the equilibrium the output of glucose by the liver and the insulin secretion, regulated by a feedback loop between hepatic cells and pancreatic β-cells (37, 38).

In comparison with fasting plasma insulin (FPI), HOMA-IR is a more accurate tool for the assessment of insulin sensitivity for several reasons. First, it gives the possibility to assess the relationship between the functioning of β-cells and insulin sensitivity in individuals with impaired glucose tolerance. Moreover, it allows to perform repeated measurements over long periods of time in subjects who continue to present abnormal glucose tolerance.

During the last decades several HOMA-IR indexes have been proposed according to the different formulas. HOMA1-IR, the original HOMA model by Matthews et al. (36), contains an easier approximation of the original non-linear solution to the interactive equations (39). HOMA2-IR is the updated computer model, which has non-linear solutions; this is the preferred model when HOMA is compared with other models, such as the minimal model. The updated of the HOMA model considers the variations in hepatic and peripheral glucose resistance^5^.

Although different studies have tried to identify normal values of this model for children and adolescents, reliable reference ranges of HOMA-IR are not available yet (40, 41). In 2015 Shashai et al. conducted a study on 2,573 Caucasian children and adolescents in order to define the specific percentiles of HOMA-IR in relation to age, gende-, and BMI and to establish suitable cut-offs to distinguish between low and high cardiometabolic risk. Their findings demonstrate that, even though age, gender and body adiposity are responsible for IR physiological changes, values higher than 1.68 in normal-weight subjects defines a “non-physiological state” and may pose the patient at an increased risk for cardiovascular disease. Instead, if subjects are overweight and obese, the cut-off rises to 3.42 (34).

The QUICKI model is like the simple equation used in the HOMA models with the exception that it is derived from the inverse of the sum of the logarithms of the fasting insulin and fasting glucose. Therefore, there is a perfect inverse correlation between QUICKI and HOMA. Thus, the disadvantages or limitations of the two methods are identical.

The main disadvantages of HOMA-IR and QUICKI are that changes occur in β-cell function over time and there is not an universal insulin assay standardization. Moreover, no data are available to prove the efficacy of markers of IR to predict response to treatment. In addition, in case of mutations of the insulin gene, the circulating insulin has subnormal bioactivity but normal immunoactivity (33, 42–44).

Some clinicians use serum sex hormone-binding globulin (SHBG) as a surrogate index of IR, but it has not been fully validated in the clinical setting, particularly in children (45).

In asymptomatic patients, serum triglyceride concentrations, and particularly the ratio of triglyceride to high-density lipoprotein (HDL) cholesterol concentrations are also useful markers for IR in children^6^.

The triglyceride-to-HDL cholesterol (TG/HDL-C) ratio has been observed to be associated with IR in white obese boys and girls. In fact, a study conducted on a population of 1,452 obese youths undertaking an oral glucose tolerance test and a fasting lipid profile, showed that the TG/HDL-C ratio is strongly related to insulin secretion and sensitivity and might therefore be used in a clinical setting in order to defined children and adolescent with insulin resistance in different ethnic groups (46).

The Mc Auley Index represents another surrogate index which has been demonstrated to have a higher sensitivity with a similar specificity in predicting insulin sensitivity if compared with fasting insulin alone, however in study mainly performed in young adults (47).

Two other insulin sensitivity markers obtained during an oral glucose tolerance test (OGTT) have been validated in adults. These indexes, which are well-correlated with the *M*-values for stimulated insulin sensitivity derived from the euglycemic-hyperinsulinemic clamp, are defined as: the whole body insulin sensitivity index (WBISI), conceived by Matsuda and De Fronzo (48), and the insulin sensitivity index (ISI), introduced by Soonthornpun et al. (49). Regarding these indexes, Yeckel et al. have demonstrated that the effects on glycemia and insulinemia derived from the OGTT in obese youths can be used to estimate insulin sensitivity and that the WBISI and ISI indexes may be potential good instruments for more complex studies evaluating insulin sensitivity in larger samples (50).

## Assessment of Risk Factors of Insulin Resistance in Children

During the last decades several studies have defined several and well-characterized risk factors for IR including ethnicity, puberty, adipose tissue variant depots, polycystic ovary syndrome (PCOS), gene variants, family history of Diabetes or gestational diabetes, and fetal growth pattern during pregnancy.

The two most important unchangeable risk factors for IR in children are ethnicity and puberty (3). Several studies have demonstrated that Caucasian children are affected by IR more often than African, American, Hispanic, Pima Indian and Asian children (51–53).

Puberty is physiologically responsible for IR; in fact, during this period of life, insulin sensitivity undergoes a decline of around 25–50% and improves when puberty ends (51–53).

There is a strong association between IR and abdominal obesity, which is known to represent one of the main elements of the MS (54). According to large-population based studies, subcutaneous adipose tissue (SAT), and visceral adipose tissue (VAT) seem to be both related to HOMA-IR. In addition, VAT correlates more strongly to insulin variables than SAT. The pathogenesis on the basis of this evident correlation is still not known exactly but several hypotheses have been considered. SAT and VAT secrete free fatty acids into blood, and higher plasmatic free fatty acid levels seem to be associated with IR. Moreover, VAT presents a strong correlation with endothelial dysfunction and higher blood C-reactive protein values, which may give an explanation to inflammation secondary to higher VAT depots.

Since adipose tissue acts as an endocrine organ, these two tissues play a fundamental role in this field. VAT is more strongly associated with adiponectin levels and releases interleukin-6 (IL-6) and plasminogen activator inhibitor-1 (PAI-1) to a greater extent than SAT. These main factors may be the cause of an higher IR in patients with elevated VAT, in fact IL-6, and PAI-1 reduce in case of weight loss and properly correlate with a parallel improvement of insulin sensitivity (55).

Adolescents affected by PCOS often present IR, whose severity has been observed to be higher in obese patients than in lean ones (56, 57).

Risk factors include genetics, which is considered a great determinant in the incidence of IR. The principal variants which increase the risk to develop type 2 DM, that could represent the final effect of IR, are listed in Table 3. The peroxisome proliferator-activated receptor gamma (PPARγ) variant Pro12Ala was one of the first genetic variants found to be related to a decreased risk of developing T2D (58–63). Gene variants of the transcription factor 7-like 2 (TCF7L2) are associated to the risk of developing diabetes more than any other gene^7^. rs972283 is located near KLF14 (kruppel-like factor 14). KLF14 gene and protein expression have been observed to be significantly decreased in both muscle and adipose tissue in individuals affected by T2D. The insulin receptor substrate 1 (IRS1) is one of the loci responsible for the insulin signaling pathway. The allele C at rs2943641 adjacent to IRS1 was found to be related to IR and hyperinsulinaemia in a European population, whereas the SNP, rs2943650, near IRS1, has found to be related to a lower percentage of body fat, higher triglycerides and IR, and decreased HDL-cholesterol. Glucokinase regulator (*GCKR*) encodes a protein which inhibits glucokinase, which is involved in the regulation glucose storage in the liver.

**Table 3:** Main gene variants associated to insulin resistance.

**SNP**	**Nearby gene**	**Locus**
rs13081389	*PPARγ*	3p25.2
rs972283	*KLF14*	7q32.2
rs2943641	*IRS1*	2q36.3
rs780094	*GCKR*	2p23.3
rs8050136	*FTO*	16q12.2
rs7903146	*TCF7L2*	10q25.2-10q25.3
rs1208	*NAT2*	8p22
rs6723108,rs998451	*TMEM163*	2q21.3
rs35767	*IGF1*	12q23.2
rs12970134	*MC4R*	18q21.32
rs17046216	*SC4MOL*	4q32.3
rs7077836	*TCERG1L*	10q26.3
rs702634, rs4311394	*ARL15*	5q11.2

Variants within the first intron of FTO gene are strongly associated with both BMI and insulin sensitivity. Mutations in N-acetyltransferase 1 (NAT1) or 2 (NAT2) impair insulin sensitivity. Low concentrations of IGF-1 in the blood sample are associated with a reduction in insulin sensitivity. Protein phosphatase 1 regulatory subunit 3B (PPP1R3B) is a recent discovered locus which seems to be involved in the glycogen synthesis in skeletal muscle, whereas growth factor receptor bound protein 14 (GRB14) has an interaction with receptor tyrosine kinases (64).

Being born from a mother with pre-existing diabetes mellitus (DM) or gestational DM (GDM) is another risk factor for obesity and impaired insulin sensitivity, even in offspring with normal birth weight (65–67). A major component of body fat has been observed in these newborns (68), although the association between the excess of adiposity and future development of IR remains controversial (3).

Even if the criteria for diagnosing GDM are absent, hyperglycaemia during pregnancy is a relevant risk factor for obesity and IR in the offspring (69).

It has been noticed that children who gain weight too fast have higher risk to develop IR in childhood and to present long-term effects of IR in adulthood (70–76).

Increased IR has been also found in preterm children, with a persistence in adulthood in association with truncal obesity (77).

## Consequences of Insulin Resistance in Children

The adverse effects of IR are primarily due to the hyperinsulinemia which occurs in case of IR (26, 27). Reduced insulin sensitivity represents an important risk factor for the development of T2D (3), being one of the two fundamental elements involved in the pathogenesis of T2D, together with β-cell dysfunction (78). IR is strongly correlated with hypertriglyceridemia and hypertension, high C-reactive protein levels, T2D and low plasma HDL-cholesterol. In addition, MS and cardiometabolic risk factors are well-known outcomes of IR in several ethnic groups (10). In fact, IR has been demonstrated to be a reliable marker in the prediction of cardiovascular risk (79, 80).

An observational study on a Japanese adult population from the Kyushu-Okinawa Population Study (KOPS) and on an American sample of adult Caucasian individuals from the Framingham Offspring Study revealed that the prevalence rate of cardiovascular diseases in Japanese population was much lower than the one observed in the USA^8^. More importantly, these differences could not be considered secondary to standard cardiovascular disease risk factors, but were related to significant population differences in IR (81).

In another study on a community-based sample exclusive of diabetes from the Framingham Heart Study, the incidence of coronary heart disease (CHD) events associated with low HDL-cholesterol (HDL-C), or low triglycerides concentrations was markedly increased only if IR was present. In contrast, if IR was absent, triglycerides or HDL-C, did not show to be significant risk factors for CHD. Moreover, this study confirmed the concept derived from more general studies that IR might represent an independent predictor of cardiovascular risk in either general populations and diabetic populations^9^ (82).

It has been experimentally shown that increase blood triglycerides concentrations, as well as decreased levels of HDL-C, can be secondary to IR; in fact, IR improved the hepatic synthesis and secretion of triglyceride-rich VLDL particles. These evidences support the concept that the risk for CHD associated with HDL-cholesterol and triglycerides may be better determined in conjunction with IR measurement. Even though tools for the measurement of fasting plasma insulin still need to be defined, it has been demonstrated that even a locally defined measure of IR, combined with decreased HDL-C and increased triglycerides concentrations, is more accurate in the identification of the CHD risk than the use of triglycerides and HDL-C blood concentrations (82)^10^.

In a community-based cohort from the Framingham Heart Study no relation between IR and incident atrial fibrillation (AF) has been observed (83). Lower natriuretic peptide levels have been registered in both non-obese and obese individuals. A relative natriuretic peptide deficiency, probably due to the IR found in obese individuals may represent one reason of the induction of hypertension by IR (84).

One of the mechanisms through which MS and IR are involved in the pathogenesis of cardiovascular diseases seems to be found in the impairment of vascular function by influencing the component metabolic abnormalities that comprise MS. In fact, in a large community-based cohort study using age- and sex-adjusted models, a relation between IR and larger baseline brachial artery diameter and higher baseline flow was observed.

The components of the MS are strictly linked to endothelial dysfunction, in particular with a reduced endothelium-dependent vasodilation. This endothelial dysfunction seems to be associated also to IR in selected patients, such as young obese individuals; in addition, insulin resistant offspring of diabetic individuals have a reduced brachial artery flow-mediated dilation. Other studies observed a lack of association between IR and vascular dysfunction when concurrent risk factors, including the MS components, were adjusted, suggesting a strong relation between the components of MS and IR in the pathogenesis of cardiovascular diseases (85).

It has been suggested that IR may be involved in the pathogenesis of atherosclerosis, according to the evidence that the more pronounced IR in youths is, the more circulating biomarkers of endothelial dysfunction are elevated, while adiponectin, which plays an antiatherogenic role, is reduced (86).

In a study conducted on the German population involved in the Kooperative Gesundheitsforschung in der Region Augsburg (KORA) S4/F4 study, lower values of adiponectin have been demonstrated to be associated with higher risk of T2D in insulin resistant individuals, estimated by homeostasis model assessment (HOMA-IR), but not in insulin sensible ones; this evidence suggests that low adiponectin may have a deleterious effect only in presence of IR. Moreover, an association between high ceramides with saturated fatty acids in the blood sample and HOMA-IR and between higher sphingomyelins with saturated fatty acids and lower HOMA-IR in individuals with normal BMI was observed. This study prompts lowering of circulating ceramides with saturated fatty acids as a possible target in pre-diabetes (87, 88).

Laboratory studies have shown that in β-cells in culture (inducing an increase of sphingomyelin by inhibiting its hydrolysis) prevents palmitate-induced lipotoxicity (89), while the inhibition of sphingomyelin synthase in myotubes (causing a reduced sphingomyelin level) leads to an impaired insulin signaling (90). Conversely, studies conducted on knock-out mice show the protective role of lowering sphingomyelin high-fat induced obesity and IR (91, 92).

Clamp studies in adolescents and young adults showed that non-alcoholic fatty liver disease (NAFLD) is associated with IR, probably because of the increased abdominal visceral adiposity (93, 94).

## Screening and Prevention

The relevant association between IR and cardiovascular risk in the pediatric population is well known, especially in obese children (95–97), even though further research is needed to clarify this association. However, the available tools for the measurement of insulin sensitivity are costly, complex and long-lasting and there is no indication for treating isolated IR (3). Therefore, the preconditions for the fulfillment of a screening project in children are absent, even in obese patients (3, 98, 99).

Among the tools proposed for the screening of IR, the insulin sensitivity index (ISI0,120), proposed by Gutt et al. may represent a useful alternative predictor of both DM and CVD events, even if it deserves further evaluation, particularly in children.

A cohort study on 2,898 from the Framingham Offspring Study without diabetes or cardiovascular diseases at baseline demonstrated that the ISI0,120 and the MS phenotype are independent predictors of CVD. In fact, as shown by the National Cholesterol Education Program (NCEP), MS phenotype seems not to be enough complete for the detection of the cardiovascular disease risk associated with IR (100)^11^.

The evidence offered by the Prevention of Renal and Vascular End Stage Disease (PREVEND) study on a Dutch cohort from the general population of the city of Groningen, suggest that the Lipoprotein Insulin Resistance Index (LP-IR) may represent a valid tool for the detection of individuals with IR, who risk the development of T2DM even if no clinical signs, such as overweight or impaired glycemia, are manifest^12^. In addition, LP-IR considers early lipoprotein alterations in insulin resistant subjects completely reversable with intervention with diet and exercise, making primary prevention easier and more efficient. Independent population studies, such as the Multi-Ethnic Study of Atherosclerosis (MESA) and the Women's Health Study (WHS), have confirmed the association between LP-IR and secondary development of T2DM, even in individuals who have the prerequisites to be considered at low risk. These studies suggest that LP-IR may add significant information to the Framingham Offspring Study (FOS) risk score due to the early occurrence of the lipoprotein changes in comparison to dysglycemia (101).

Primary prevention of IR consists in avoiding the most common changeable risk factors including maternal obesity, gestational diabetes, maternal undernutrition and smoking during pregnancy (65, 102–106). Moreover, although a direct association between breast-feeding and improved insulin sensitivity is unknown, breast-feeding should be promoted because it is involved in preventing obesity in children (107, 108).

Finally, physical activity should be promoted in all children, in association with combined intervention programs aimed at avoiding excessive weight gain (3).

## Treatment

The first approach to IR in children consists of lifestyle interventions, including dietary modifications and increased physical activity.

Some studies suggest that exercise may have a major impact on the improvement of insulin sensitivity than the isolated reduction of body mass index (109). The Framingham Heart Study gives evidence that there is a significant association between physical activity and sedentary time and insulin sensitivity and adipokine blood levels. In particular, physical activity is associated with higher insulin sensitivity, while sedentary time is positively associated with leptin and fatty acid binding protein (FABP) levels. In addition, it has been observed that the number of steps per day is directly related to IGF-1 levels and inversely related to high sensitivity C-reactive protein (hsCRP) values.

Despite the evidence of the role of exercise training programmes in the improvement of insulin sensitivity, the mechanisms through which physical activity reduces this pathological condition and whether this response is simply the effect of changes in body composition is still unknown (110).

It has been demonstrated that a reduced fat intake through diet improve insulin sensitivity in adolescents (3, 111). In fact, a diet based on a high intake of whole-grain and fibers seems to promote weight loss and lower IR (112).

A pharmacologic intervention in obese children is sometimes needed to implement the effects of these primary prevention interventions. However, rare but serious side effects have been observed with all the available medications, therefore they should be used in selected cases. Patients' age, weight and comorbidities should be listed when considering pharmacological therapies and a close monitoring is needed once they are introduced. Moreover, longer-term (≥1 year) evidence of benefits in youth is still missing (113, 114).

Metformin, a biguanide derivate, has been demonstrated to have beneficial results in further decreasing BMI (115). It reduces insulin resistance by decreasing fasting plasma glucose and insulin concentrations in adults, as demonstrated in large, randomized, clinical trials. Metformin has been approved by the US Food and Drug Administration for the treatment of T2D in 10-year-old or older children and it is the only treatment evaluated in formal clinical trials concerning prediabetes in children. In non-diabetic obese adults it reduces food intake, causing weight loss and reduction of fasting plasma glucose, cholesterol, and insulin concentrations. In addition, metformin has been demonstrated to improve BMI, body fat composition, fasting glucose, insulin, glycated hemoglobin (HbA1c), IR expressed by the HOMA-IR, blood pressure and lipid profile in short trials conducted on small sample sizes of children and adolescents (115)^13^. Although it has been observed that metformin improves insulin sensitivity in adolescents with T2D and PCOS, metformin is not yet indicated as a treatment for isolated IR (3).

Long-term and consistent data are still missing to establish its role in the pediatric population.

Gastro-intestinal adverse drug reactions, among which abdominal pain, nausea, metallic taste, bloating, and diarrhea, are commonly observed in patients treated with metformin and can be prevented or intensely reduced by starting its administration with low doses and increasing the dose gradually or using extended-release formulations.

Infrequent side effects observed in clinical trials are lactic acidosis, especially in case of overdose, and vitamin B12 deficiency, requiring monitoring, and when necessary adequately supplementing this vitamin when necessary.

In conclusion, metformin is safe and presents evident positive effects on insulin sensitivity, but it should be introduced only in selected patients, as suggested by the 2017 Pediatric Obesity Clinical Guidelines from The Endocrine Society. Furthermore, its long-term benefits in insulin-resistant children are still to be analyzed.

Glucagon-like peptide-1 (GLP-1) is a gut hormone, which interferes with the inflammatory processes by reducing the release of inflammatory cytokines and inhibiting the infiltration of macrophages into the adipose tissue, the liver and the blood vessel wall (116). Since chronic inflammation is involved in the pathogenesis of IR, the pharmacodynamics studies conducted on GLP-1, suggest that GLP-1 analogs, such as Liraglutide, could improve insulin sensitivity in insulin resistant patients (116). Danne et al. (117) conducted a 5 week randomized, double-blind, placebo-controlled trial on 21 obese adolescents from 12 to 17 years old and with a Tanner stage of 2 to 5. Gastrointestinal side effects of liraglutide, especially abdominal pain, were observed in 96.5% of cases and mild hypoglycaemia occurred in 8 patients, in one-half of cases after prolonged fast. No severe hypoglycaemia was reported. No severe treatment emergent adverse effects were observed.

Despite the favorable effects of liraglutide, such as improvement of BMI z score, body weight, FPG, HbA1c and fasting serum insulin observed, none was statistically significant, probably due to the short duration of the trial and small number of participants (117).

Dipeptidylpeptidase-4(DPP4) inhibitors are novel oral glucose-lowering drugs which decreases the inhibition of endogenous incretins to induce the secretion of insulin in relation to glucose blood levels, resulting in improved fasting plasma glucose, postprandial glucose, and HbA1c level. Although there is no evidence in children, DPP4 inhibitors, in combination with insulin, have been demonstrated to decrease HbA1c blood concentrations with a minor weight gain and incidence of hypo- glycemia in adults affected by T2D (118).

The sodium–glucose cotransporter type 1 (SGLT1) is the main transporter involved in the absorption of glucose and galactose in the gastrointestinal tract, while sodium–glucose cotransporter type 2 (SGLT2) is located in the kidney, where it is responsible for the reabsorption of 90% filtered glucose (119, 120). Phase 2 and 3 clinical studies conducted on Sotagliflozin, an oral potent dual inhibitor of SGLT1 and SGLT2, have shown an improved glycaemic control with lower HbA1c, less side effects such as body weight and hypertension, and a persistent efficacy in lower estimated glomerular filtration rate levels in adults with type 1 and 2 diabetes. However, more consistent data are needed to establish its real benefits and side effects on insulin-resistant adults and children (120).

Weight loss drugs such as sibutramine and orlistat are able to improve insulin sensitivity in children and adolescents, although their use need to be properly and selectively adopted in this age group (121–123).

The management of congenital generalized lipodystrophy is multidisciplinary and must be adapted to the characteristics of each patient. It may include psychological support, cosmetic surgery, the promotion of a high carbohydrate, low-fat diet and regular exercise for type 1 CGL, the avoidance of strenuous exercise and use of β-adrenergic blockers and other anti-arrhythmic medications for patients with type 4 CGL. It is unclear whether patients with type 2 CGL and cardiomyopathy should avoid exercise (15). For subjects who present extremely high blood triglycerides levels treatment with fibric acid derivatives are suggested, adding, in specific cases, low-dose statins in order to reduce non-HDL cholesterol.

Metformin and sulphonylureas are the first-line therapy for diabetes mellitus in patients with CGL.

Metreleptin, a recombinant analog of human leptin, seems to be effective in the treatment of metabolic complications in type 1 and 2 CGL. This molecule has its main effect on hypothalamus reducing appetite. In 2014, the FDA approved the use of metreleptin, in addition to dietary changes, in patients with CGL and AGL (15).

The only therapy available for patients with leprechaunism to date is recombinant IGF1, aiming to prevent compensatory hyperinsulinemia (23). IGF has about the 6% of the glucose-lowering action effect of insulin and has a similar structure to it, therefore it is able to bind to insulin receptors triggering peripheral glucose uptake and glycogen synthesis and inhibiting protein catabolism. The efficacy of this treatment is still controversial and further studies are needed but evidence form the small number of *in vivo* studies available at the moment seems promising (23).

## Conclusions

IR is a pathological condition strongly associated with obesity and involved in the pathogenesis of T2D. Obesity is not the only determinant of IR; prolonged use of corticosteroids or growth hormone therapy and genetic diseases may be responsible for this condition too.

The gold standard for the assessment of IR is the hyperinsulinemic euglycemic clamp, nevertheless its costs and difficult management in clinical and research activity have determined the need of surrogate methods. HOMA-IR and QUICKI present a favorable correlation with the hyperinsulinemic-euglycemic clamp, however they are not considered a valid test for the evaluation of IR, therefore in the clinical practice the diagnosis of IR in obese patients is based on clinical features, including hyperglycemia, dyslipidemia, abdominal obesity, and hypertension. The main unchangeable risk factors for IR in children are the Caucasian ethnicity and puberty. At present, the preconditions for the fulfillment of a screening project in children are absent, even in obese patients. The prevention of IR in children consists in avoiding maternal obesity, gestational diabetes, maternal undernutrition, and smoking during pregnancy and encouraging breast-feeding and physical activity.

The treatment of insulin resistant children is firstly targeted to lifestyle interventions. In selected cases, the integration of a pharmacological intervention is needed. However, adequate data concerning the safety and long-term efficiency of drugs in patients with IR are not available yet.

Despite several studies have been conducted in children and adolescent with IR, many questions remain open. Further studies, and above all large-population long-lasting observational studies should be undertaken in order to unravel the unsolved issues related to its pathogenesis, diagnosis and treatment.

## Author Contributions

All the authors have made a substantial, direct and intellectual contribution to the work. CF revised it critically. All authors listed approved the work for publication.

### Conflict of Interest Statement

The authors declare that the research was conducted in the absence of any commercial or financial relationships that could be construed as a potential conflict of interest.

## Footnotes

^1^Available online at: www.ncbi.nlm.nih.gov (accessed January 5, 2019).

^2^Available online at: www.science.gov (accessed January 15, 2019).

^3^Available online at:https://www.pl.scribd.com

^4^Available online at: https://www.ucsfhealth.org/tests/003466.html (accessed May 2, 2019).

^5^Available online at: https://www.care.diabetesjournals.org (accessed January 10, 2018).

^6^Available online at: https://www.diabetesincontrol.com (accessed January 20, 2019).

^7^Available online at: https://www.helmholtz-muenchen.de (accessed January 20, 2019).

^8^Available online at: https://www.jcb.rupress.org (accessed January 20, 2010).

^9^Available online at: https://www.atvb.ahajournals.org (accessed January 8, 2019).

^10^Available online at: https://www.atvb.ahajournals.org (accessed January 8, 2019).

^11^Available online at: https://www.diabetes.diabetesjournals.org (accessed January 15, 2019).

^12^Available online at: https://www.pdfs.semanticscholar.org (accessed January, 15 2019).

^13^Available online at: https://www.eprints.ucl.ac.uk (accessed January 1, 2019).
